# The Hidden Psychological Burden of Burn Injury: Clinical Characteristics Correlation of Psychiatric Disorders in Hospitalized Burn Patients at the Burn Unit of Dr. Cipto Mangunkusumo National General Hospital

**DOI:** 10.3390/ebj7030042

**Published:** 2026-08-11

**Authors:** Feranindhya Agiananda, Aditya Wardhana, Artasya Karnasih, Natasya Ayusandra Mahersaputri

**Affiliations:** 1 Consultation-Liaison Psychiatry Division, Department of Psychiatry, Faculty of Medicine, Universitas Indonesia, Dr. Cipto Mangunkusumo National General Hospital, Jakarta 10430, Indonesia; 2 Burn and Wound Care Division, Department of Plastic Surgery and Reconstructive Surgery, Faculty of Medicine, Universitas Indonesia, Dr. Cipto Mangunkusumo National General Hospital, Jakarta 10430, Indonesia

**Keywords:** burn injury, clinical characteristics, psychiatric disorder

## Abstract

**Highlights:**

**What are the main findings?**

- Among the 201 patients with burns included in this study, 73.1% were diagnosed with a psychiatric disorder, with adjustment disorder being the most prevalent. Female sex, multiple psychosocial stressors, and more than three operative procedures were identified as independent predictors.
- Despite 83.1% of patients sustaining major burns, the burn area represented by Total Body Surface Area (TBSA) was not an independent predictor of psychiatric disorder, likely due to a ceiling effect from the near-universal psychiatric morbidity observed across all injury severity categories.

**What are the implications of the main findings?**

- The cumulative burden of repeated operative procedures, rather than the severity of anatomical injury, is a key driver of psychiatric risk in patients with burns, warranting targeted psychiatric screening in patients undergoing complex surgical management.
- The routine integration of psychiatric-liaison services into multidisciplinary burn care is strongly recommended. Where a universal psychiatric assessment is not feasible, a stepped screening approach may help prioritize patients requiring further evaluation, particularly women and those experiencing multiple concurrent psychosocial stressors or repeated operative procedures.

**Abstract:**

**Background:** Psychiatric disorders are common but often unrecognized complications in patients with burns. This study aimed to determine the association between psychiatric disorders and demographic and clinical factors in the Burn Unit of Dr. Cipto Mangunkusumo National General Hospital (RSCM). **Methods:** This retrospective cross-sectional study used the electronic medical records (EMR) of adult patients with burns admitted between 2023 and 2025. **Results:** Of the 201 inpatients with burns, 73.1% were diagnosed with a psychiatric disorder, most commonly adjustment disorder (53.2%); among patients with major burns, this rose to 83.1%. Female sex (odds ratio (OR) = 0.421; *p* = 0.032), multiple concurrent psychosocial stressors (OR = 0.389; *p* = 0.012), and more than three operative procedures (OR = 0.127; *p* = 0.010) were independent predictors. There were no independent links between burn extent (Total Body Surface Area) and psychiatric outcomes, or between length of stay (LOS) and psychiatric outcomes. A significant proportion of inpatients with burns had psychiatric disorders. **Conclusions:** The risk of psychiatric morbidity was significantly associated with female sex, multiple psychosocial stressors, and an increased operative burden, but not with burn severity. These results support regular psychiatric screening as an integral part of the comprehensive treatment of patients with burns, especially in high-risk groups.

## 1. Introduction

Burn injuries may result in a substantial psychological burden in addition to physical injury. They are often unexpected, can be very harmful, and can leave the person with a traumatizing experience [1]. This can occur due to exposure to heat, cold, chemicals, or electricity. Thermal mechanisms (flames, hot liquids, and heated solid materials) cause the most burns worldwide today [1,2].

Burns are estimated to contribute to between 7 and 12 million injuries and 180,000 deaths annually, and are a significant public health problem, especially in low- and middle-income countries [3]. A previous study conducted at Dr. Cipto Mangunkusumo National General Hospital (RSCM) indicated that the majority of burn injuries were caused by flames (54.9%), scalds (29.2%), and electrical (12.0%) or chemical exposure (3.1%) [4]. Furthermore, the latest research by the RSCM indicated that the majority of patients with burns had second and third degree burns on the skin surface area, which encompassed more than 60% of their total body surface area (TBSA), and were injured by fire in 67.9% of the cases. Importantly, 57.1% of patients reported psychological symptoms, and significant negative associations were found between psychological symptoms and quality of life domains [5].

Although burn care has improved and burn survival rates for patients with burns have risen, patients with burns have a higher long-term morbidity, impacting their quality of life and causing higher disability-adjusted life years (DALYs). Physical changes such as scars, contractures, and amputation may also occur, resulting in permanent disability in everyday functioning and intimate relationships, social stigma, and persistent pain in most cases. As a result, the psychological effects of burn injury have become more of a focus [3,6].

Patients with burns have been found to be more vulnerable to mental health problems than the general public. Van Dam et al. identified acute stress reactions, adjustment disorders, and depressive disorders as the predominant psychiatric conditions among patients with burns, with a higher prevalence observed in females than in males [7]. Other studies have found that psychiatric disorders were the most common among patients with burns, occurring in about 54%, of which nearly 45% were major depressive disorders, with social anxiety disorder and post-traumatic stress disorder (PTSD) also commonly found within this demographic [8]. In a cohort of patients with severe burns, approximately 60% reported experiencing anxiety symptoms during follow-up [9]. A recent systematic review of studies conducted during the first 12 months after a burn injury found that anxiety symptoms ranged from 6% to 49%, and the formal diagnosis of PTSD ranged from 4.4% to 22% in the severe burn group [7].

The distribution of psychiatric diagnoses among patients with burns should also be interpreted in relation to their temporal diagnostic criteria. The International Classification of Diseases, 10th Revision (ICD-10) specifies that acute stress reactions typically emerge within hours of an exceptional stressor and resolve within days, rarely persisting beyond one month, whereas adjustment disorders require onset within one month of a stressor and usually resolve within six months. By contrast, PTSD may develop after a latency period ranging from several weeks to months after a traumatic event. Consistent with these temporal patterns, survivors of burn injury have been shown to most commonly develop PTSD three to six months after injury, with depressive symptoms increasing in prevalence over time [10]. Understanding these time-dependent diagnostic windows provides an important context for interpreting the distribution of psychiatric diagnoses observed during acute hospitalization.

Multiple factors related to the burn injuries and treatment processes contribute to the development of psychiatric disorders in patients with burns. A scoping review suggested that the severity of burns, longer hospitalization, and use of invasive procedures are related to the psychological consequences. Patients with severe burns are at a higher risk for post-burn psychiatric sequelae, likely due to the systemic complications and invasive treatments that accompany more extensive injuries. Pais et al. also noted that patients with burns treated in the Intensive Care Unit (ICU) are at a heightened risk of psychological disorders after hospitalization, partly due to Post-Intensive Care Syndrome (PICS) and prolonged hospital stays [11,12]. Furthermore, comorbid chronic conditions such as diabetes mellitus, cardiovascular disease, chronic pulmonary disease, and substance use disorders may exacerbate psychological outcomes through mechanisms related to inflammation, neuroendocrine function, and neurotransmitter activity. Psychological distress can be exacerbated by several surgical interventions (e.g., debridement, necrotic tissue removal, and skin grafting), pain associated with surgery, and dependence on opioid analgesics and sedatives after surgery [13].

Although these risks have been acknowledged, studies examining the demographic and clinical factors associated with psychiatric disorders in Indonesian patients with burns remain limited. This study aimed to determine the associations between sociodemographic and clinical characteristics and the presence of psychiatric disorders among patients admitted to the Burn Unit of RSCM. The findings could inform the development of multidisciplinary burn care protocols that incorporate psychosocial screening and early psychiatric interventions.

## 2. Materials and Methods

### 2.1. Participant and Study Design

This was a retrospective observational study with a cross-sectional analytical design to evaluate the association between demographic and clinical characteristics of burn injury and psychiatric disorders in patients with burn injury. Psychiatric assessment was mandated by the institutional protocol for all patients admitted to the Burn Unit of RSCM during hospitalization, regardless of burn severity, conducted in structured interviews based on ICD-10 diagnostic criteria, exclusively by certified psychiatrists. The data were collected from the electronic medical records (EMR) of patients with burns admitted to the Burn Care Unit of the RSCM from January 2023 to December 2025. This study was approved by the Ethics Committee of the Faculty of Medicine, Universitas Indonesia (KET-506/UN2.F1/ETIK/PPM/00.02/2026), and was reported in accordance with the Strengthening the Reporting of Observational Studies in Epidemiology (STROBE) guidelines (Table S1).

A total of 201 participants were included in the study. Consecutive samples were used, and all the patients who met the inclusion criteria were included until the desired sample size was reached. Patients were included if they were adults (age 18 years and above), remained conscious during their inpatient stay, and had complete EMR data. Patients who died before completing their treatment in the Burn Care Unit, exhibited persistent impairment of consciousness that hindered psychiatric evaluation during hospitalization, were discharged against medical advice prior to finishing the care episode, and/or had incomplete medical records were excluded from the study.

### 2.2. Data Collection

The main outcome variable was the presence or absence of psychiatric disorders during hospitalization. The independent variables were the demographic and clinical variables. The demographic factors included age, sex, marital status, and psychosocial stressors. Clinical characteristics specific to patients with burns included TBSA categorized into minor-to-moderate (<20%) or major (≥20%, including any burns involving the face, hands, feet, genitalia or major joints [14]); the visibility of the burn injury, which was classified as either invisible (wounds concealed by clothing) or visible; pain intensity, assessed using the Numeric Rating Scale (NRS) and categorized into mild (1–3), moderate (4–6), or severe (7–10); LOS, which was dichotomized into short (≤16 days) or long (>16 days) based on the median; admission to the ICU of the Burn Unit; etiology of the burn injury; delay between injury and hospital admission (burn onset) (≤1 day or >1 day); chronic disease comorbidities; and the number of operative procedures (none, 1–3, or >3).

### 2.3. Data Analysis

Analysis of the data gathered from the EMR was performed using the IBM Statistical Package for the Social Sciences (SPSS) version 27 (IBM Corp., Armonk, NY, USA). All variables were analyzed for descriptive statistics using a univariate analysis. All data are presented as frequencies and percentages, except for the continuous variable age, which is expressed as mean ± standard deviation. Spearman’s correlation test was used for continuous variables, and the chi-square test, Fisher’s exact test, or Fisher–Freeman–Halton exact test was used for categorical variables, as appropriate, for the bivariate analysis. Statistical significance was set at *p* < 0.05. Variables that showed statistical significance in the bivariate analysis were then entered into a binary logistic regression model for multivariate analysis to determine those that were independent predictors of psychiatric disorders. The Hosmer–Lemeshow goodness–of–fit test was performed to assess the fit of the model. Odds ratios (OR) and 95% confidence intervals (95% CI) are given.

## 3. Results

**Figure 1:**
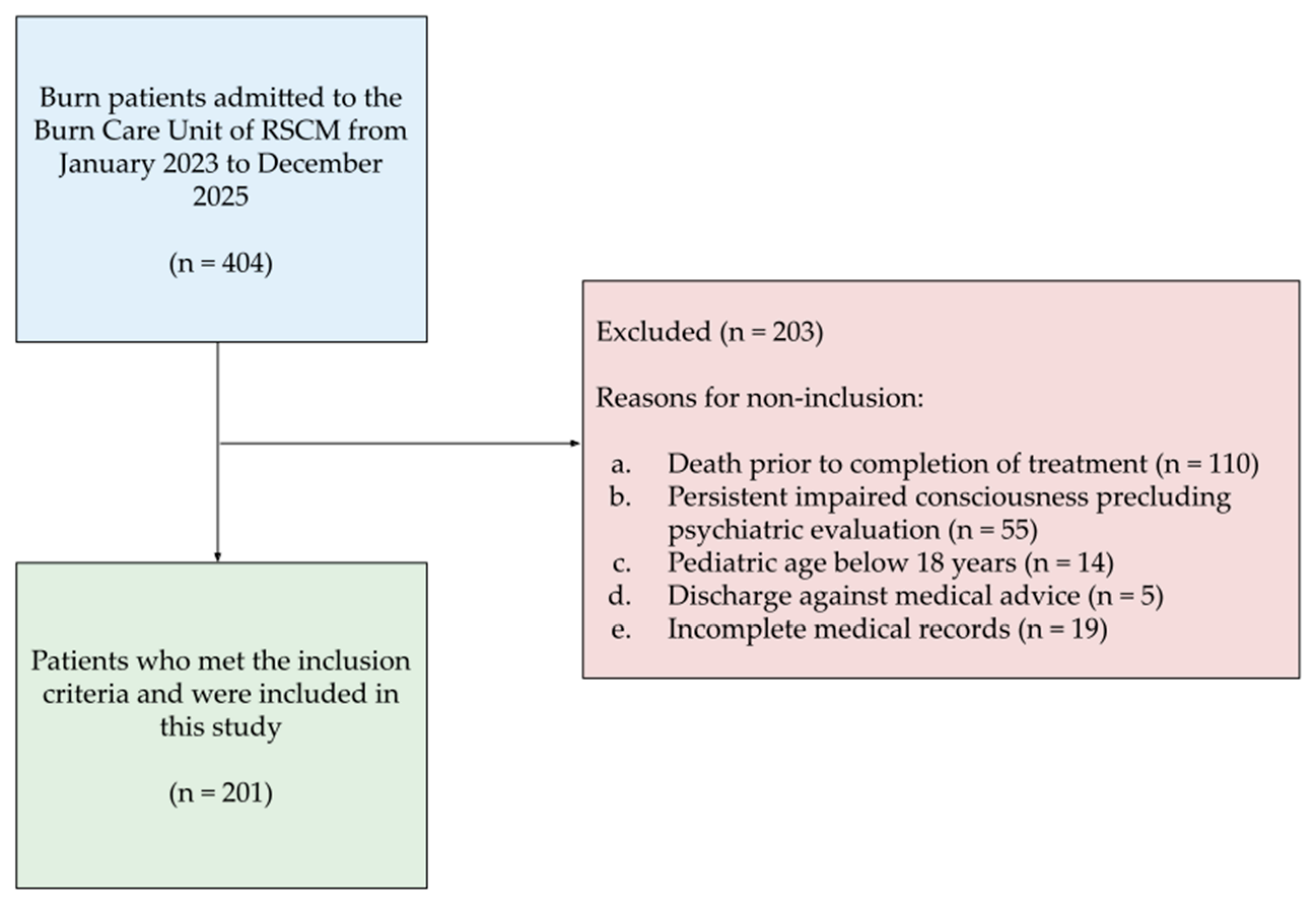
Flow diagram of participant selection process, showing the number of burn patients admitted, reasons for non-inclusion, and the final study sample.

Of the 404 burn patients admitted to the Burn Care Unit of RSCM during the study period, 201 met the inclusion criteria and were included in the final study sample. The participant selection process, including the reasons for non-inclusion, is summarized in Figure 1.

This study used consecutive sampling to collect the data. To assess the representativeness of the study sample, demographic and clinical characteristics of the study sample were compared with those of the total population of burn patients admitted during the study period, as shown in Table 1 below:

The study sample did not differ significantly from the total admitted population in terms of age (*p* = 0.103) or sex distribution (*p* = 0.699), suggesting that the consecutive sampling approach used in this study did not introduce substantial demographic selection bias. However, the study sample showed a significantly longer length of stay (LOS > 16 days; *p* < 0.001) and a significantly lower proportion of major burns (TBSA ≥ 20%; *p* = 0.003) compared with the total admitted population. The clinical basis for these differences, and their implications for the generalizability of the study findings, are addressed in the Section 4.

### 3.1. Univariate Analysis

Descriptive analysis was used to summarize the study population of 201 patients, based on demographic and clinical parameters, as presented in Table 2.

Participants’ mean age was 36.73 years (SD ± 11.40). The majority of the patients were male (*n* = 133; 66.2%), and most were married (*n* = 138; 68.7%). Among psychosocial stressors, the most common were the current medical condition (*n* = 106; 52.7%) and multiple concurrent stressors (*n* = 87; 43.3%), summarized in Table 3.

A psychiatric disorder was diagnosed in many of the patients (*n* = 147, 73.1%). Most of the diagnoses recorded were adjustment disorder (*n* = 107, 53.2%), acute stress reaction (*n* = 25, 12.4%), depressive disorder (*n* = 7, 3.5%), and PTSD (*n* = 7, 3.5%). The other was also diagnosed as having Alcohol Dependence (currently abstinent in hospital) (0.5%).

From a clinical standpoint, most of the patients suffered from major burns (*n* = 167; 83.1%), and 95% of them had a visible wound. Most of them were hospitalized for a long period of time (*n* = 113; 56.2%), and moderate pain was most commonly reported (*n* = 92; 45.8%). Flame burns constituted 73.6% of cases (*n* = 148). The majority of patients were treated in the High Care Unit (HCU) (67.7%, *n* = 136). There were 17.9% (*n* = 36) of patients with chronic comorbid diseases. Most of them (*n* = 133; 66.2%) had received one to three surgical procedures while in hospital, and 60.2% (*n* = 121) had been seen in hospital within 1 day of their burn injury.

### 3.2. Bivariate Analysis

A bivariate analysis was conducted to examine the associations between each independent variable and psychiatric disorders. Spearman’s correlation test was used for age, and Chi-square or Fisher–Freeman–Halton exact tests (Monte Carlo method) were used for categorical variables. The psychiatric diagnosis was recoded as either “no psychiatric disorder” or “psychiatric disorder present” before the analysis. In addition, the cause of the burn was also divided into “electrical burns” and “non-electrical burns”. Results of the bivariate analysis are presented in Table 4.

LOS (*p* = 0.003), psychosocial stressor (*p* = 0.009), sex (*p* = 0.015), and number of operative procedures (*p* < 0.001) were the variables that correlated statistically with the occurrence of psychiatric disorders. Conversely, the variables that did not achieve statistical significance were burn area or TBSA (*p* = 0.136), pain intensity (*p* = 0.390), marital status (*p* = 0.458), age (*p* = 0.893), burn etiology (*p* = 0.138), ward location (*p* = 0.875), chronic disease comorbidity (*p* = 0.488), burn onset (*p* = 0.418), and burn wound visibility (*p* = 0.730).

### 3.3. Multivariate Analysis

To identify factors independently associated with psychiatric disorders, a binary logistic regression analysis was performed. The overall model was statistically significant (*p* < 0.001) and showed a good fit (*p* = 0.910) according to the Hosmer-Lemeshow test, as shown in Table 5.

Males were associated with decreased odds of psychiatric disorder compared with females (OR = 0.421; 95% CI: 0.191–0.929; *p* = 0.032). The number of surgical procedures was also significantly related to psychiatric disorders. Patients with no surgical interventions had significantly lower odds than those with more than 3 surgical interventions (OR = 0.127; 95% CI: 0.026–0.609; *p* = 0.010). Moreover, there was no statistically significant association between patients undergoing 1–3 procedures and lower odds (OR = 0.472; 95% CI: 0.186–1.198; *p* = 0.144).

Regarding psychosocial stressors, psychiatric disorders were significantly less likely to occur in patients with a medical condition as their major stressor (OR = 0.389; 95% CI: 0.186–0.812; *p* = 0.012) than in those with multiple co-occurring stressors. However, Length of Stay (LOS) (OR = 0.617; 95% CI: 0.294–1.295; *p* = 0.202) and burn severity measured by TBSA (OR = 0.645; 95% CI: 0.262–1.585; *p* = 0.339) were not found to be independently associated with psychiatric disorder.

## 4. Discussion

This study aimed to assess and explore the prevalence and factors associated with psychiatric disorders in patients with burns admitted to the Burn Unit of RSCM between 2023 and 2025. It was concluded that there was a high psychological burden associated with burn injuries, with psychiatric disorders present in 73.1% of patients with burns. The prevalence observed in this study was higher than the typical range of 30–60%, perhaps due to the setting of a national tertiary general hospital burn center, where patients have more severe burns and a more complex treatment pathway [15,16]. Moreover, psychiatric disorders were identified using careful psychiatric assessment through structured interviews by a certified psychiatrist while the patients were hospitalized, and were not solely based on symptom screening tools.

The most prevalent diagnosis was adjustment disorder, representing 53.2% of cases, reflecting the acute psychosocial turmoil that accompanies hospitalization, physical disfigurement, pain, and prolonged treatment. This finding was consistent with a previous study in consultation-liaison psychiatry environments, which showed that adjustment disorder was identified in 60% or more of burn patients who were referred to psychiatry, establishing it as the most frequently diagnosed psychiatric condition in this clinical setting [17,18]. Acute stress reaction was the second most common diagnosis, identified in 12.4% of patients with burns, reflecting the immediate traumatic situation associated with the burn injury. The relatively low rates for PTSD (3.5%) and depressive disorder (3.5%) may be due to the timing of psychiatric evaluations during hospitalization, with psychiatric disorders being more likely to emerge or become more apparent with time. In addition, the duration of acute inpatient care in the burn unit might have been insufficient to meet the diagnostic threshold for residual post-traumatic and/or depressive symptoms. This is consistent with a systematic review and meta-analysis of 32 studies reporting a pooled prevalence of PTSD of 20.5% in survivors of burn injury within the first two years after burn injury [7].

Women were found to be independently associated with an increased risk of psychiatric disorders (OR = 0.421; *p* = 0.032), aligning with studies showing that women are approximately two to three times more susceptible than men to developing stress-related and affective psychopathologies after experiencing traumatic events [19,20]. Gender differences in emotional processing, coping skills, perception of disfigurement, and/or sociocultural expectations have been suggested as potential mechanisms underlying women’s increased risk of stress-related psychopathologies such as psychiatric disorders after traumatic events. Furthermore, women tend to feel threatened to their physical image and social identity, increasing psychological distress after the occurrence of a burn injury that is visible to others [21].

A higher number of surgical procedures was also significantly associated with the presence of psychiatric disorders, as those who had more than three procedures were the most vulnerable (OR = 0.127 for no procedure vs. >3, *p* = 0.010). This is especially interesting, considering the high proportion of psychiatric disorders in this group. The psychological effects of repeated surgeries, such as procedural anxiety, pain, prolonged periods of inactivity, and anxiety and distress during anesthesia, may be more important for psychiatric morbidity than the anatomical severity of the injury. The repeated nature of surgical interventions for burn injury, such as skin grafting, debridement, and reconstructive surgery in patients with burns can also present a high psychiatric risk due to the cumulative impact of the successive events of repeated surgery, stress, and re-traumatization that each individual experiences [22].

Although nearly 75% of the patients had sustained a major burn, TBSA was not independently associated in the multivariate model (OR = 0.645; *p* = 0.339). This observation undermines the notion that the risk of psychological effects is necessarily higher in situations involving larger burns. This suggests that the severity of burn may not be sufficient to explain the psychiatric morbidity in patients with burns in the hospital. A possible explanation for this finding is that there was a high prevalence of psychiatric disorders throughout this study, which may have made it difficult for TBSA to successfully predict psychiatric outcomes. In contrast, the number of surgical procedures is a variable and cumulative component of the clinical course and is likely to be markedly different, even among patients with large burns. Our findings are consistent with those of previous studies that showed an inconsistent relationship between TBSA and psychiatric outcomes [23]. In 2021, a longitudinal study found that TBSA was correlated with body image dissatisfaction, but not directly with symptoms of PTSD, suggesting that psychological outcomes may be mediated by psychosocial factors depending on the severity of injury. Likewise, a prospective cohort study from 2025 found that TBSA is an ambiguous predictor of post-burn PTSD, yielding mixed results across various studies, especially when considering non-injury-related factors [24,25].

Patients with more than one concurrent stressor had an increased risk of psychiatric disorders compared to those with medical stressors as their main stressor (OR = 0.389; *p* = 0.012). This observation aligns with diathesis-stress models, which propose that the accumulation of stressors can surpass an individual’s ability to cope, resulting in psychopathology [26]. The findings support the importance of psychosocial assessment in burn care, particularly for patients with additional financial, caregiving, work, or social difficulties. LOS was found to be significantly related in bivariate analysis (*p* = 0.003) but not in multivariate analysis (*p* = 0.202). This means that LOS may not be a psychological risk factor but is a measure of clinical severity and operational complexity. After controlling for other covariates and surgical procedures, LOS was no longer a predictor of psychiatric disorders.

Other factors, including burn etiology, pain severity, burn visibility, ward location, comorbidities, and timing of burn onset, were not significantly correlated with psychiatric disorders. Given the psychological implications of scarring and disfigurement, the lack of importance of burn visibility was surprising. This may be explained by the cross-sectional design of our study, as distress related to disfigurement can intensify during the inpatient rehabilitation period after hospital discharge [24]. Similarly, although pain intensity has been associated with long-term depression and PTSD in survivors of burn injury, it may have been masked by the uniform use of analgesic protocols in the burn unit [27].

The results highlight the importance of integrating consultation-liaison psychiatric services in the multidisciplinary care of burn patients, irrespective of injury severity. Early psychiatric engagement may allow for timely identification of psychological distress, support adaptive coping, and improve psychosocial support during hospitalization. Particular attention should be paid to patients with the risk factors identified in this study, including female sex, multiple concurrent psychosocial stressors, and repeated operative procedures. However, universal assessment by consultation-liaison psychiatry services may not be feasible in healthcare settings with limited specialist resources. Comprehensive psychiatric assessment using a structured interview may also require considerable time and may be difficult to conduct during the acute phase of burn treatment when patients frequently experience pain, fatigue, physical discomfort, or limited tolerance for prolonged evaluation. In such settings, a stepped assessment approach may provide a more feasible alternative. Standardized screening instruments such as the Self-Reporting Questionnaire-29 (SRQ-29) may initially be used to screen mental and emotional problems, while the Patient Health Questionnaire-9 (PHQ-9) may be used specifically to screen for depressive symptoms [28,29]. Patients with positive screening results should subsequently undergo a comprehensive psychiatric interview to establish a formal diagnosis and determine the need for appropriate psychiatric treatment and follow-up care. This stepped approach may reduce the assessment burden during acute hospitalization while facilitating the early identification and appropriate referral of patients requiring specialist psychiatric care.

There are several limitations to this research. First, the retrospective cross-sectional design limits the ability to make temporal or causal inferences between clinical characteristics and psychiatric disorders. Second, the study was conducted in a single tertiary national burn center, and the results may not be generalizable to other burn centers or populations with different burn severities or access to psychiatric services. Longitudinal and/or multicenter studies would be helpful in examining the development of psychiatric disorders after the acute hospitalization phase, during rehabilitation, and over a long period in the course of recovery, particularly in patients receiving multiple surgical procedures and in those experiencing very strong psychosocial stressors. Third, the study sample differed significantly from the total admitted population in terms of LOS and burn severity, although age and sex distributions were similar. This pattern may be explained by the exclusion of patients who died before treatment completion or had persistent impaired consciousness, together comprising the majority of exclusions. These groups were disproportionately represented among those with more extensive burns, consistent with the established association between TBSA and burn mortality [30,31]. The shorter LOS in these more severely injured patients likely resulted from early death rather than a milder clinical course, as burn-related mortality is concentrated in the first one to two weeks after injury [32]. This represents an inherent selection limitation arising from the eligibility criteria—specifically, the requirement for sustained consciousness to complete a structured psychiatric interview—rather than a flaw in the sampling methodology, as consecutive sampling was applied uniformly to all eligible patients. Consequently, our findings may not be generalizable to the most severely injured burn patients, who were unable to participate in psychiatric evaluation and may have experienced a psychological burden not captured in this study. Fourth, both LOS and TBSA were analyzed exclusively as categorical variables (LOS: ≤16 vs. >16 days; TBSA: minor-moderate vs. major) rather than as continuous measures. While dichotomization facilitates clinical interpretability and aligns with commonly used severity thresholds in burn literature, and dichotomizing a continuous variable at the median is also reliable, this approach is known to reduce statistical power. Future studies analyzing LOS and TBSA as continuous variables or using a larger number of clinically meaningful categories may help to further clarify their relationship with psychiatric outcomes in patients with burns.

## 5. Conclusions

This study showed that psychiatric disorders are common in burn units, and adjustment disorder was the most common diagnosis. Female sex, multiple psychosocial stressors, and the number of surgical procedures were independently associated with psychiatric disorders, but burn severity (TBSA) was not independently associated with psychiatric morbidity. These findings highlight the significant psychological stress experienced by patients during acute burn treatment and the importance of including psychiatric evaluation in a multidisciplinary burn care program. Patients who underwent multiple surgeries and/or have multiple psychosocial stressors should be targeted. Identification of psychological stress in the early phase of hospitalization can support psychosocial adaptation and help to improve the comprehensive care of patients with burns. In addition, these results support the importance of integrating consultation-liaison psychiatry services into a multidisciplinary approach for burn care units.

## Figures and Tables

**Table 1:** Comparison of Demographic Characteristics Between the Study Sample and the Total Population of Burn Patients Admitted During the Study Period (January 2023–December 2025).

Characteristics	Study Sample (*n* = 201)	Total Population (*n* = 404)	*p*-Value
Age (mean ± SD)	36.73 ± 11.40	37.75 ± 12.32	0.103
Sex, *n* (%)			0.699
Male	133 (66.2)	269 (66.6)	
Female	68 (33.8)	135 (33.4)	
Burn Area or TBSA, *n* (%)			**0.003**
Minor-Moderate	34 (16.9)	48 (12.1) *	
Major	168 (83.1)	348 (87.9) *	
LOS, *n* (%)			**<0.001**
Short (≤16 days)	88 (43.8)	212 (54.5) **	
Long (>16 days)	113 (56.2)	177 (45.5) **	

SD = Standard Deviation; TBSA = Total Body Surface Area; LOS = Length of Stay. * LOS data were available for 389 of 404 patients (15 records incomplete). ** TBSA data were available for 396 of 404 patients (8 records incomplete). Variables that are significant at <0.05 are bolded.

**Table 2:** Demographic Characteristics of Burn Patients Treated in the Burn Unit of RSCM, 2023–2025.

Characteristic	Category	*n*	%
Age (mean ± SD)	36.73 ± 11.40	201	–
Sex	Male	133	66.2
	Female	68	33.8
Marital Status	Married	138	68.7
	Unmarried	55	27.4
	Divorced	8	4.0
Psychosocial Stressor	Current Medical Condition	106	52.7
	Financial Problems	3	1.5
	Family/Caregiver Support	5	2.5
	Multiple Stressors	87	43.3

SD = standard deviation. *n* = 201.

**Table 3:** Clinical Characteristics of Burn Patients Treated in the Burn Unit of RSCM, 2023–2025.

Characteristic	Category	*n*	%
Psychiatric Disorder	No disorder	54	26.9
	Adjustment Disorder	107	53.2
	Acute Stress Reaction	25	12.4
	Depressive Disorder	7	3.5
	Post-Traumatic Stress Disorder (PTSD)	7	3.5
	Alcohol Dependence (abstinent)	1	0.5
Burn Area or TBSA	Minor–Moderate	34	16.9
	Major	167	83.1
Burn Wound Visibility	Invisible wounds only	10	5.0
	Visible wounds present	191	95.0
LOS	Short (≤16 days)	88	43.8
	Long (>16 days)	113	56.2
Pain Intensity (in categorized NRS)	Mild (1–3)	60	29.9
	Moderate (4–6)	92	45.8
	Severe (7–10)	49	24.4
Burn Etiology	Flame burn	148	73.6
	Scald burn	28	13.9
	Electrical burn	22	10.9
	Flash burn	1	0.5
	Chemical burn	1	0.5
	Contact burn	1	0.5
Ward Location	High Care Unit	136	67.7
	Intensive Care Unit	65	32.3
Chronic Disease Comorbidity	None	165	82.1
	Present	36	17.9
Number of Operative Procedures	None	13	6.5
	1–3 procedures	133	66.2
	>3 procedures	55	27.4
Burn Onset	≤1 day	121	60.2
	>1 day	80	39.8

TBSA = Total Body Surface Area; LOS = Length of Stay; NRS = Numeric Rating Scale, *n* = 201.

**Table 4:** Bivariate Analysis: Association of Variables with Psychiatric Disorder Occurrence.

Variable	*p*-Value
Burn Area or TBSA	0.136
**LOS**	**0.003**
Pain Intensity (in categorized NRS)	0.390
Marital Status	0.458
**Psychosocial Stressor**	**0.009**
**Sex**	**0.015**
Age	0.893
Burn Etiology	0.138
Ward Location	0.875
Chronic Disease Comorbidity	0.488
**Number of Operative Procedures**	**<0.001**
Burn Onset	0.418
Burn Wound Visibility	0.730

TBSA = Total Body Surface Area; LOS = Length of Stay; NRS = Numeric Rating Scale. Significant *p*-values (<0.05) are bolded.

**Table 5:** Multivariate Logistic Regression Analysis of Factors Associated with Psychiatric Disorder in Burn Patients.

Variable	Comparison	OR (95% CI)	*p*-Value
Sex	Male vs. Female	**0.421 (0.191–0.929)**	**0.032**
LOS	Short vs. Long	0.617 (0.294–1.295)	0.202
Psychosocial Stressor	Medical condition vs. Multiple stressors	**0.389 (0.186–0.812)**	**0.012**
	Financial vs. Multiple stressors	0.477 (0.035–6.485)	0.578
	Social support vs. Multiple stressors	1.029 (0.097–10.890)	0.981
Burn Area or TBSA	Minor-moderate vs. Major	0.645 (0.262–1.585)	0.339
Number of Operative Procedures	None vs. >3	**0.127 (0.026–0.609)**	**0.010**
	1–3 vs. >3	0.472 (0.186–1.198)	0.144

LOS = Length of Stay; TBSA = Total Body Surface Area; OR = Odds Ratio; CI = Confidence Interval. Reference categories: Female (sex); Long stay (LOS); Multiple stressors (psychosocial); >3 operative procedures; Major burn (TBSA). Independent variables that are significant at <0.05 are bolded.

## Data Availability

The dataset supporting the findings of this study is not publicly available due to privacy and ethical considerations. However, de-identified data may be accessible upon reasonable request to the corresponding author, subject to review and approval by the relevant compliance and ethics authorities at Dr. Cipto Mangunkusumo National General Hospital (RSCM).

## Supplementary Materials

The following supporting information can be downloaded at: https://www.mdpi.com/article/10.3390/ebj7030042/s1, Table S1: STROBE Statement—Checklist of items that should be included in reports of cross-sectional studies.

## Abbreviations

The following abbreviations are used in this manuscript:

CI	Confidence Interval
EMR	Electronic Medical Records
LOS	Length of Stay
NRS	Numeric Rating Scale
OR	Odds Ratio
ICU PHQ-9	Intensive Care Unit Patient Health Questionnaire-9
PICS	Post-Intensive Care Syndrome
PTSD	Post-Traumatic Stress Disorder
RSCM	Dr. Cipto Mangunkusumo National General Hospital
SD	Standard Deviation
SPSS	Statistical Package for Social Sciences
SRQ-29	Self-Reporting Questionnaire-29
STROBE	Strengthening the Reporting of Observational Studies in Epidemiology
TBSA	Total Body Surface Area

## References

[1] Gautam R., Rajoura O.P., Sharma A.K., Bhatnagar R., Srivastava R. (2022). Socio-demographic features and quality of life post burn injury. J. Fam. Med. Prim. Care.

[2] Jeschke M.G., Kamolz L.P., Sjöberg F., Wolf S.E. (2019). Epidemiology of burns. Handbook of Burns.

[3] Gerstl J.V., Ehsan A.N., Lassarén P., Yearley A., Raykar N.P., Anderson G.A., Smith T.R., Sabapathy S.R., Ranganathan K. (2024). The global macroeconomic burden of burn injuries. Plast. Reconstr. Surg..

[4] Martina N.R., Wardhana A. (2013). Mortality analysis of adult burn patients. J. Plast. Rekons..

[5] Winanda R.A., Wardhana A., Irawati D.R. (2017). The association between psychopathology and quality of life in burn patients at Dr. Cipto Mangunkusumo Hospital, Jakarta. J. Plast. Rekonstr..

[6] Lodha P., Shah B., Karia S., De Sousa A. (2020). Post-traumatic stress disorder (PTSD) following burn injuries: A comprehensive clinical review. Ann. Burn. Fire Disasters.

[7] Boersma-van Dam E., Shepherd L., van de Schoot R., Engelhard I.M., Van Loey N.E.E. (2025). The prevalence of posttraumatic stress disorder symptomatology and diagnosis in burn survivors: A systematic review and meta-analysis. Health Psychol. Rev..

[8] Kadam K.S., Bagal R.P., Angane A.Y., Ghorpade G.S., Anvekar A.R., Unnithan V.B. (2021). A cross-sectional study of quality of life, psychiatric illness, perceived social support, suicidal risk and self-esteem among patients with burns. J. Fam. Med. Prim. Care.

[9] Panayi A.C., Heyland D.K., Stoppe C., Jeschke M.G., Didzun O., Matar D., Tapking C., Palackic A., Bliesener B., Harhaus L. (2024). The long-term intercorrelation between post-burn pain, anxiety, and depression: A post hoc analysis of the RE-ENERGIZE trial. Crit. Care.

[10] World Health Organization (1992). The ICD-10 Classification of Mental and Behavioural Disorders: Clinical Descriptions and Diagnostic Guidelines.

[11] Pais M.A., Vasella M., Matthes O., Millesi E., Kobler A., Breckwoldt T., Reid G., Naef L., Hofmann L., Watson J.A. (2025). Severe burn injuries and the impact of mental health: Insights from 7 years at Switzerland’s leading burn center. Intern. Emerg. Med..

[12] Obed D., Schroeter A., Gruber L., Salim M., Krezdorn N., Vogt P.M. (2023). Outcomes following burn injury in intensive care patients with major psychiatric disorders. Burns.

[13] Paggiaro A.O., Paggiaro P.B.S., Fernandes R.A.Q., Freitas N.O., Carvalho V.F., Gemperli R. (2022). Posttraumatic stress disorder in burn patient: A systematic review. J. Plast. Reconstr. Aesthetic Surg..

[14] Lee N., Bae Y., Jang S., Lee D.W., Lee S.W. (2025). Global, Regional, and National Burden of Burn Injury by Total Body Surface Area (TBSA) Involvement from 1990 to 2021, with Projections of Prevalence to 2050. Healthcare.

[15] Wang S., Cannata B., Vallurupalli M., Yenikomshian H.A., Gillenwater J., Stoycos S.A. (2024). A scoping review of PTSD and depression in adult burn patients: A call for standardized screening and intervention research. J. Burn Care Res..

[16] Spronk I., Legemate C.M., Beerthuizen A., Van Loey N.E., Van Baar M.E., Polinder S. (2023). Physical, functional, and psychosocial recovery from burn injury are related and their relationship changes over time: A Burn Model System study. Burns.

[17] Lotzin A., Stahlmann K., Acquarini E., Ajdukovic D., Ajdukovic M., Anastassiou-Hadjicharalambous X., Ardino V., Bondjers K., Bragesjö M., Böttche M. (2024). A longitudinal study of risk and protective factors for symptoms of adjustment disorder during the COVID-19 pandemic. Eur. J. Psychotraumatol..

[18] Kofman O., Kofman E. (2022). Adjustment disorder: Current developments and future directions. Int. J. Environ. Res. Public Health.

[19] Seedat S., Halliday S. (2023). Sex-based contributors to and consequences of post-traumatic stress disorder. Curr. Psychiatry Rep..

[20] Haering S., Schulze L., Geiling A., Meyer C., Klusmann H., Schumacher S., Knaevelsrud C., Engel S. (2024). Higher risk—Less data: A systematic review and meta-analysis on the role of sex and gender in trauma research. J. Psychopathol. Clin. Sci..

[21] Gavrilova Y., Donevant J., Ficalora J., Kahn S.A. (2024). Effect of gender on risk of PTSD/depression and symptom outcomes 30-days post-injury in burn patients. J. Burn Care Res..

[22] Wang Y.Q., Wu Z.H., Chen X.J., Ma H. (2023). Patient-reported outcomes and their predictors 2 years after burn injury: A cross-sectional study. Int. Wound J..

[23] Rehan M., Tariq R., Iqbal T., Sarwar M.A., Tul Ain Q., Waheed U. (2024). Impact of burns on anxiety, depression and SELFESTEEM among patients with burn injuries: A cross-sectional study. Ann. Burn. Fire Disasters.

[24] Huang Y.K., Su Y.J. (2021). Burn severity and long-term psychosocial adjustment after burn injury: The mediating role of body image dissatisfaction. Burns.

[25] Ford D., Waller M., Das A., Cameron C.M., Warren J., Druery M. (2025). Baseline predictors of depression and post-traumatic stress disorder (PTSD) symptoms in hospitalized adult burn survivors: A longitudinal, prospective cohort study. Burns.

[26] Bjørndal L.D., Kendler K.S., Reichborn-Kjennerud T., Ystrom E. (2023). Stressful life events increase the risk of major depressive episodes: A population-based twin study. Psychol. Med..

[27] Lewis J., Pride L.C., Lee S., Anwaegbu O., Tabukumm N.N., Patel M.M., Lee W.-C. (2024). Examining the role of post-traumatic stress disorder, chronic pain and opioid use in burn patients: A multi-cohort analysis. Scars Burn Heal..

[28] Qatrunnada R.Z., Suseno B., Yazid M. (2025). Psychometric analysis of the self-reporting questionnaire (SRQ-29) among university students. J. Ilm. Psikol. Terap..

[29] Kroenke K., Spitzer R.L., Williams J.B.W. (2001). The PHQ-9: Validity of a brief depression severity measure. J. Gen. Intern. Med..

[30] Wardhana A., Leksono T.P., Sanjaya I.S., Handiansyah B.S., Farhana N. (2026). Association of total body surface area on effective surgical treatment and mortality in adult burn patients: A 5-year retrospective analysis in an Indonesian National Referral Hospital. Turk. J. Emerg. Med..

[31] Lip H.T.C., Idris M.A.M., Imran F.H., Azmah T.N., Huei T.J., Thomas M. (2019). Predictors of mortality and validation of burn mortality prognostic scores in a Malaysian burns intensive care unit. BMC Emerg. Med..

[32] Yoneda K., Osuka A., Ohnishi S., Matsuura H., Oda J. (2024). The timing of death in burn patients. Acute Med. Surg..

