# Supplementary material for: The Hidden Psychological Burden of Burn Injury: Clinical Characteristics Correlation of Psychiatric Disorders in Hospitalized Burn Patients at the Burn Unit of Dr. Cipto Mangunkusumo National General Hospital
Source: Eur Burn J. 2026 Aug 11;7(3):42. doi: 10.3390/ebj7030042 (PMC13510477; doi:10.3390/ebj7030042)
Supplement: Supplementary file 1 [file ebj-07-00042-s001.zip › ebj-4424503-supplementary.pdf]

Table S1. STROBE Statement—Checklist of items that should be included in reports of ***cross-sectional studies***

	Item No	Recommendation	Reported on page/section
**Title and abstract**	1	(*a*) Indicate the study's design with a commonly used term in the title or the abstract	Abstract; Methods §2.1 ("This was a retrospective observational study with a cross-sectional analytical desgin…")
		(*b*) Provide in the abstract an informative and balanced summary of what was done and what was found	Abstract
**Introduction**			
Background/ rationale	2	Explain the scientific background and rationale for the investigation being reported	Introduction, paras 1-6
Objectives	3	State specific objectives, including any prespecified hypotheses	Introduction, para 7
**Methods**			
Study design	4	Present key elements of study design early in the paper	Methods §2.1, para 1
Setting	5	Describe the setting, locations, and relevant dates, including periods of recruitment, exposure, follow-up, and data collection	Methods §2.1, para 1-2 (adult patients admitted to the Burn Care unit of RSCM from January 2023 – December 2025, using databases from Electronic Medical Record of RSCM.
Participants	6	(*a*) Give the eligibility criteria, and the sources and methods of selection of participants	Methods §2.1, para 2 (inclusion/exclusion criteria); consecutive sampling with mandatory psychiatric assessment protocol for all admitted Burn Care unit of RSCM patients.
Variables	7	Clearly define all outcomes, exposures, predictors, potential confounders, and effect modifiers. Give diagnostic criteria, if applicable	Methods §2.2
Data sources/ measurement	8*	For each variable of interest, give sources of data and details of methods of assessment (measurement). Describe comparability of assessment methods if there is more than one group	Methods §2.2 – All datas resourced from the Electronic Medical Record of RSCM. The demographic datas resourced from the registration procedure before patients admitted to the Burn Care Unit. Clinical diagnoses resourced from assessment from plastic surgeons. Psychiatric diagnoses established via structured clinical interviews by certified psychiatrist, per institutional protocol mandating psychiatric assessment for all admitted patients in Burn Care Unit.
Bias	9	Describe any efforts to address potential sources of bias	Adressed via Methods: (1) Additional information of mandatory psychiatric assessment protocol, and Appendix: (2)

			Empirical comparison of sample vs total population demographic profiles (Table A1) showing no significant difference in age (p=0.103) and sex (p=0.699)
Study size	10	Explain how the study size was arrived at	Methods §2.1 states sample size = 201. Sample size was calculated using Slovin's formula with 5% margin of error.
Quantitative variables	11	Explain how quantitative variables were handled in the analyses. If applicable, describe which groupings were chosen and why	Methods §2.2-2.3<br>Age was analysed as a continuous variable (mean ± SD). Three clinical variables were categorised for analysis. TBSA was dichotomised into minor-to-moderate (<20%) and major (≥20%), consistent with the binary TBSA-based severity classification used in recent global burden-of-disease estimates for burn injury (Lee et al., 2025). LOS was dichotomised into short (≤16 days) and long (>16 days) based on the median. Pain intensity (NRS) was categorised into mild (1–3), moderate (4–6), and severe (7–10), consistent with standard NRS severity bands.
Statistical methods	12	(*a*) Describe all statistical methods, including those used to control for confounding	Methods §2.3<br>Descriptive: presented as frequencies and percentages, except for the continuous variable age (presented as mean and SD)<br>Bivariate: Spearman's correlation test for continuous variables, Chi-square test, Fisher's exact test, or Fisher–Freeman–Halton exact test for categorical variables.<br>Multivariate: Binary logistic regression model for the statistically significant variables in the bivariate analysis (p<0.05)
		(*b*) Describe any methods used to examine subgroups and interactions	No subgroup/interaction analyses described
		(*c*) Explain how missing data were addressed	Missing data (patients with incomplete medical records) were excluded during eligibility screening (§2.1). Consequently, the included sample (n=201) had complete data for all variable of interest.
		(*d*) If applicable, describe analytical methods taking account of sampling strategy	Not applicable (using consecutive sampling)
		(*e*) Describe any sensitivity analyses	No sensitivity analyses were conducted
**Results**			

Participants	13*	(a) Report numbers of individuals at each stage of study—eg numbers potentially eligible, examined for eligibility, confirmed eligible, included in the study, completing follow-up, and analysed	Methods §2.1 / Results §3.1: "A total of 404 burn patients admitted to the Burn Care Unit of RSCM between January 2023 and December 2025, of whom 201 met the inclusion criteria and were included in this study.<br>Reasons for non-inclusion of the remaining 203 patients were: death prior to completion of treatment (n=110), persistent impaired consciousness precluding psychiatric evaluation (n=55), pediatric age below 18 years (n=14), and discharge against medical advice (n=5). The remaining patients (n=19) were excluded due to incomplete medical records, reflecting the inherent limitation of electronic medical records usage.
		(b) Give reasons for non-participation at each stage	See Item 13(a) above — reasons for non-participation reported by category
		(c) Consider use of a flow diagram	Narrative reporting used above instead given the categorical breakdown is fully reported in text
Descriptive data	14*	(a) Give characteristics of study participants (eg demographic, clinical, social) and information on exposures and potential confounders	Results §3.1, Tables 1-2
		(b) Indicate number of participants with missing data for each variable of interest	Methods §2.1, As patients with incomplete medical records were excluded during eligibility screening (see Participants, §2.1), all 201 patients included in the final analysis had complete data for every variable of interest.
Outcome data	15*	Report numbers of outcome events or summary measures	Results §3.1, Table 2
Main results	16	(*a*) Give unadjusted estimates and, if applicable, confounder-adjusted estimates and their precision (eg, 95% confidence interval). Make clear which confounders were adjusted for and why they were included	Results §3.2-3.3, Tables 3-4
		(*b*) Report category boundaries when continuous variables were categorized	Methods §2.2 (category cut-offs defined); Results Table 2
		(*c*) If relevant, consider translating estimates of relative risk into absolute risk for a meaningful time period	Not applicable
Other analyses	17	Report other analyses done—eg analyses of subgroups and interactions, and sensitivity analyses	No additional analyses beyond bivariate and multivariate analyses conducted.
**Discussion**			
Key results	18	Summarise key results with reference to	Discussion, opening paragraph

		study objectives	
Limitations	19	Discuss limitations of the study, taking into account sources of potential bias or imprecision. Discuss both direction and magnitude of any potential bias	Discussion, limitation paragraph:<br>(1) Retrospective design (limits the ability to make temporal or causal inferences between clinical characteristics and psychiatric disorders)<br>(2) Study conducted in single-center (tertiary health facility)<br>(3) LOS/TBSA analysed only as categorical variables
Interpretation	20	Give a cautious overall interpretation of results considering objectives, limitations, multiplicity of analyses, results from similar studies, and other relevant evidence	Discussion
Generalisability	21	Discuss the generalisability (external validity) of the study results	Discussion, limitation paraghraph:<br>The study was conducted at a single tertiary national burn center, and the results may not generalizable to other primary or secondary care settings which managing predominantly minor-to moderate burns.
**Other information**			
Funding	22	Give the source of funding and the role of the funders for the present study and, if applicable, for the original study on which the present article is based	This study received no external funding. Funded by the author.

*Give information separately for exposed and unexposed groups.

Note: An Explanation and Elaboration article discusses each checklist item and gives methodological background and published examples of transparent reporting. The STROBE checklist is best used in conjunction with this article (freely available on the websites of PLoS Medicine at http://www.plosmedicine.org/, Annals of Internal Medicine at http://www.annals.org/, and Epidemiology at http://www.epidem.com/). Information on the STROBE Initiative is available at www.strobe-statement.org.
